# Description of the Uncinate Process: A Computed Tomography Cross-Sectional Study

**DOI:** 10.1055/s-0044-1791259

**Published:** 2025-01-10

**Authors:** Mohammad Waheed El-Anwar, Mohamed Kamel Alawady, Ashraf El-Hussiny, Mohamed Talaat Albasiouny, Hany Alloush, Hoda Ismail Abdelhamid

**Affiliations:** 1 Department of Otorhinolaryngology, Head and Neck Surgery, Faculty of Medicine, Zagazig University, Zagazig, Egypt; 2 Department of Otorhinolaryngology, Faculty of Medicine, Al-Azhar University, Cairo, Egypt; 3 Department of Otorhinolaryngology, Faculty of Medicine, Ain Shams University, Cairo, Egypt; 4 Department of Radiodiagnosis, Faculty of Medicine, Al-Azhar University, Cairo, Egypt

**Keywords:** nose, uncinate process, middle meatus, endoscopic sinus surgery, CT

## Abstract

**Introduction** The uncinate process (UP) is the most important and constant landmark in the ostiomeatal complex and the middle meatus.

**Objective** To identify the UP variations that have not been published before and establish a categorization using computed tomography (CT).

**Methods** The current study was carried out on 110 paranasal CT scans (220 sides). Axial images were acquired with multiplanar reformats to capture delicate details in other planes.

**Results** Out of 120 CT scans (220 sides), the UP was found to be of type 1 in 84.5%, type 2 in 12.3%, and type 3 in 3.2%, without significant diferences between genders, and it was found to be medialized in 81.9%, vertical in 16.3%, lateralized in 0.9%, and absent in 0.9%, without significant differences between genders. A total of 8.63% of the UPs were pneumatized.

**Conclusion** The present study improves surgeons' and radiologists' knowledge of the UP, while creating a standard classification and description to be used as a common language between otorhinolaryngologists and radiologists, which could also be used for training.

## Introduction

Currently, endoscopic sinonasal surgery (ESS) is among the most commonly conducted otorhinolaryngology surgeries, 1 2 3 with the evolution in sinuscope technology, equipment, and imaging. 4 5 6 7 Proper imaging detail is a tool that could be used to perform an effective and safe ESS. 8 , Computed tomography (CT) is of outstanding importance to assess the sinonasal diseases and to identify the anatomic nasal variations 7 9 that could differ significantly even between the sides in the same subject. 4 7 9 10

The ostiomeatal complex (OMC) is a key region of the lateral nasal wall that represents the main route for ventilation and drainage of the paranasal sinuses. Anatomic variations could obstruct this region, leading to sinuses infections, by disturbing their drainage and/or ventilation. 11

The uncinate process (UP) is the most essential and constant landmark in the OMC, and the middle meatus, and it represents a key landmark and important access area for ESS. 11 It looks like a soft, bony sickle-shaped structure that is part of the ethmoid bone and attaches to the ethmoid process of the inferior turbinate. 12

It is necessary and important to take into consideration the clinical and surgical relevance of UP variations within the OMC, which can be best observed in the coronal plane on CT. 13

Preoperative determination of anatomic UP variations on CT scans helps avoid intraoperative complications, such as injury to the medial orbital wall, nasolacrimal duct, sphenopalatine vessels, and skull base; thus, it is crucial to ESS. 14 15

Even though the UP Has BEEN studied before in the literature, most studies 16 17 focus only on the superior attachment of the UP and its relation to the frontal drainage pathway and frontal sinus surgery. However, other variations of the UP, regarding the direction, appearance and pneumatization have been sparsely described in the literature. In addition, there is still a lack of published articles collecting and classifying UP variations in detail.

Therefore, the objective of the present study was to determine the variable description, variations and types of the UP that have not been published before. The study results could contribute to the performance of safe and effective endoscopic sinonasal procedures.

## Methods

The present cross-sectional analysis was performed on 110 sinonasal CT scans (220 sides) at the Otorhinolaryngology and Radiodiagnosis Departments of University Hospitals from November 2022 to November 2023. An informed consent form was signed by all participants after a discussion of the purposes of the study, and ethical approval was obtained (IRB 68/21-jAN-2024).

The study followed the ethical principles for medical research involving human subjects of the Declaration of Helsinki. Subjects younger than 20 years of age, with history of facial trauma, sinonasal surgery, or subjects presenting neoplasms, congenital anomalies, and/or sinonasal fibro-osseous lesions were excluded from the study.

A radiological evaluation was performed using the GE LightSpeed VCT, 64-slice multidetector CT (MDCT) scanner (GE HealthCare Technologies, Inc., Chicago Il, United States) with a 0.625-mm detector width, 1.5-mm section width, and 0.5-mm interval reconstruction.

For the paranasal sinuses, axial cuts were taken with the beam parallel to the hard palate while the subjects were in the supine position, staring from the hard palate to the frontal sinus, applying 130 KV and 150 mA/seconds with 1.5 second of scan time. The scans were performed with bone window setting of 3,000 HU, at 700 HU. A high-resolution algorithm was used to improve the quality of the fine bone detail.

Multiplanar reconstructions with fine detail in all planes were acquired at a dedicated postprocessing workstation (Advantage Windows Volume Share 4.5, GE HealthCare Technologies, Inc.). Films were red in a routine standard way to not miss any detail.

**Fig. 1:**
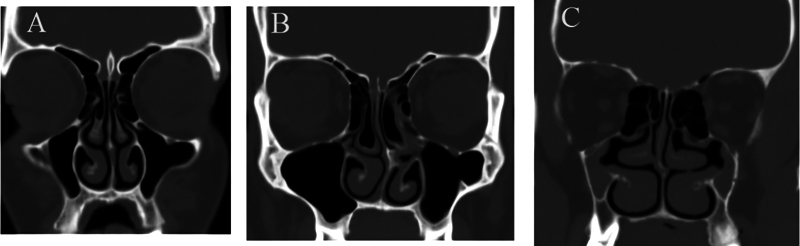
Computed tomography scans showing the types of uncinate process (UP); ( **A** ) type 1, in which the UP and the infundibulum are fully developed and defined; ( **B** ) type 2, in which there is hypoplasia of the UP and ill-defined infundibulum; and ( **C** ) type 3, in which the UP is absent.

The UP was classified into type 1, in which the UP and the infundibulum are fully developed and defined; type 2, in which there is hypoplasia of the UP and ill-defined infundibulum; and type 3, in which the UP is absent ( Fig. 1 ).

**Fig. 2:**
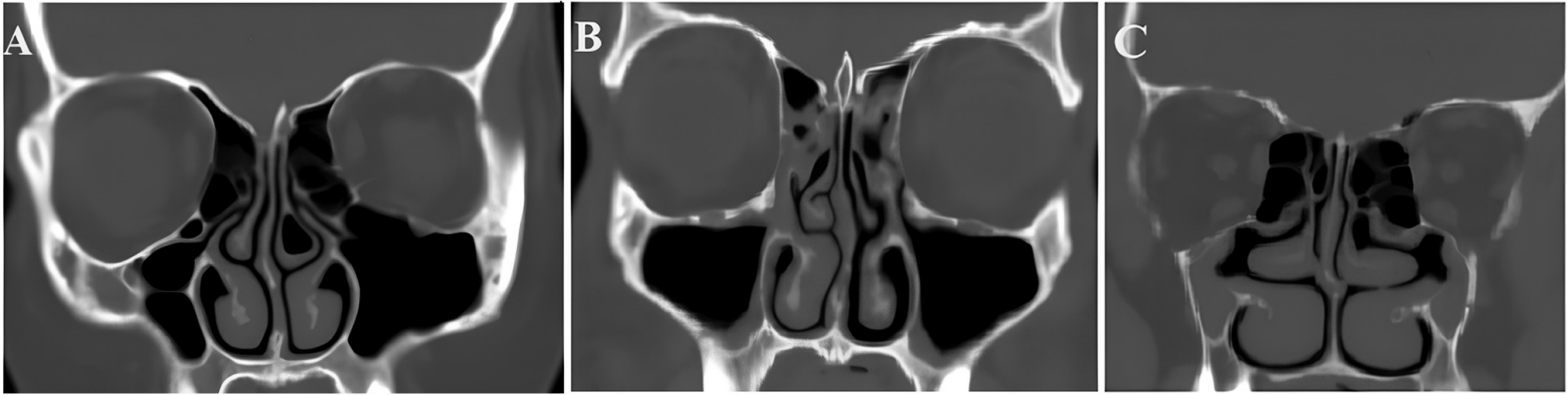
Computed tomography scans showing different directions of the UP: ( **A** ) medialized UP; ( **B** ) vertical UP; and ( **C** ) lateralized UP.

Then, the UP was evaluated according to its direction in relation to the vertical plane and classified into medialized, vertical, or lateralized ( Fig. 2 ).

**Fig. 3:**
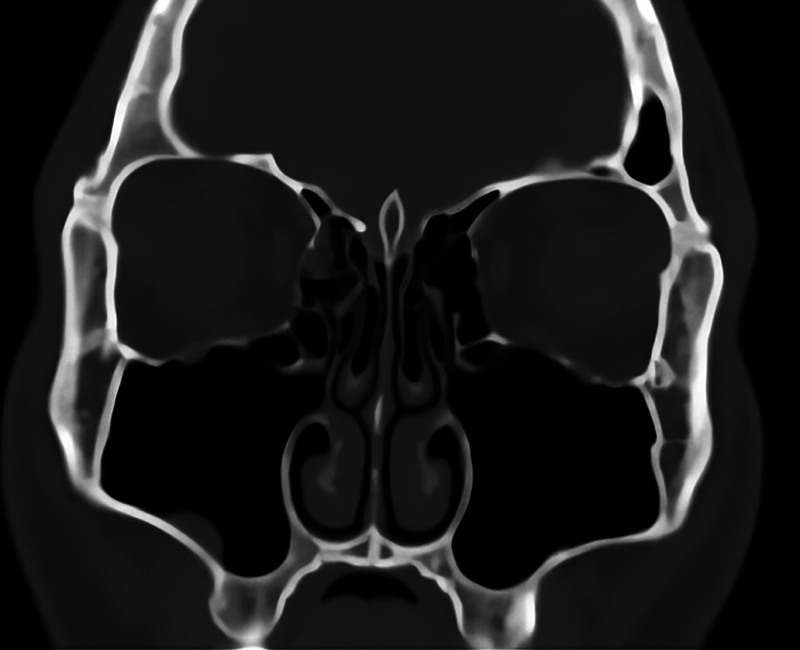
Computed tomography scan showing a pneumatized UP.

Then, the pneumatization of the UP was checked ( Fig. 3 ), and we evaluated the relation of the pneumatization of present to the nearby pneumatization at the middle meatus area. The relation of pneumatized UP to the deviation of the nasal septum was also registered.

The IBM SPSS Statistics for Windows software, version 25.0 (IBM Corp., Armonk, NY, United States) was used to perform the statistical analysis. Values of *p* < 0.05 were considered statistically significant.

## Results

The study included 110 CT scans (220 sides) of 72 male (65.5%) and 38 female patients (34.5%). Their mean age was of 34.5 ± 10.4 (range: 20–80) years.

Out of 220 sides analyzed, the UP was found to be of type 1 in 186 (84.5%) of type 2 in 27 (12.3%), and of type 3 in 7 (3.2%). Among the female patients (76 sides), the UP was of type 1 in 60 (78.9%) of type 2 in 12 (15.8%), and of type 3 in 4 (5.3%). Among the male subjects (144 sides), the UP was of type 1 in 126 (87.5%) of type 2 in 15 (10.4%), and of type 3 in 3 (2.1%). Type 1 was the most common among male and female patients, without significance differences between genders ( *p* = 0.2; Chi-squared [χ ^2^ ] = 3.181) ( Table 1 ).

**Table 1:** Types of uncinate process among the study sample

	Type 1		Type 2		Type 3		*p* -value
	Number	Percentage (%)	Number	Percentage (%)	Number	Percentage (%)	
**All sides**	186/220	84. 5	27/220	12.3	7/220	3.2	
**Male patients**	126/144	87.5	15/144	10.4	3/144	2.1	0.2. (Chi-squared = 3.181)
**Female patients**	60/76	78.9	12/76	15.8	4/76	5.3	

The UP was found to be medialized in 180 out of 220 sides (81.9%), vertical in 36 (16.3%), lateralized in 2 (0.9%), and absent in 2 (0.9%) sides. Among the female patients (76 sides), it was medialized in 61 (80.3%), vertical in 13 (17.1%), lateralized in 0 case, and absent in 2 (2.6%). Among the male patients (144 sides), the UP was medialized in 119 (82.7%), vertical in 23 (16%), and lateralized in 2 (1.4%), with no cases of absent UP. The most common type among all patients was the medialized UP. There was no significant difference between genders ( *p* = 0.1779; χ ^2^ = 4.918) ( Table 2 ).

**Table 2:** Direction of the uncinate process among the study sample

	Medialized		Vertical		Lateralized		Absent		*p* -value
	Number	Percentage (%)	Number	Percentage (%)	Number	Percentage (%)	Number	Percentage (%)	
**All sides**	180/220	81.9	36/220	16.3	2/220	0.9	2/220	0.9	
**Male patients**	119/144	82.6	23/144	16	2/144	1.4	0	0	0.1779 (Chi-squared = 4.918)
**Female patients**	61/76	80.3	13/76	17.1	0	0	2/76	2.6	

The UP was found to be pneumatized in 19 out of 220 sides (8.63%); pneumatization was detected in 11 out of 144 sides (7.64%) among male subjects, and in 8 out of 76 sides (10.52%) among female patients, without significant differences between genders (χ ^2^ = 0.526; *p* = 0.468) ( Table 3 ).

**Table 3:** Pneumatization of the uncinate process among the study sample

	Number	Percentage (%)	*p* -value
**All sides**	19/220	8.63	
**Male patients**	11/144	7.64	0.46 (Chi-squared = 0.526)
**Female patients**	8/76	10.52	

The relation between UP pneumatization and deviated nasal septum was found to be on the right side in 3/3 (100%) of male subjects and in 2/3 (66.6%) female patients.

The relation between UP pneumatization and deviated nasal septum on the left side was not found among the male subjects, with 6 out of 8 cases (75%) deviating to the right side; among the female patients, 1/5 (20%) and no deviation in septum to left side in 4/5 (80%), and septal deviation to the opposite side in 3/5 (60%).

There were other types of pneumatization in 73.6% (19) of the cases, in the form of Haller cell in 7 (36.8%), concha bullosa in 7 (36.8%), and no other pneumatization in 5 (26.4%).

## Discussion

Most paranasal sinuses drain into the OMC, 2 so ESS normally targets this area, with the UP being considered an indispensable landmark guide during ESS and part of any procedure involving the middle meatus. Thus, radiologists and ESS surgeons should be fully aware of the UP details shown on the CT scans and speak a common language in order to perform safe and effective ESS procedures.

The present study included patients older than 20 years as the the maxillary sinuses reach maturity at ∼ 20 years of age, after the development of the permanent teeth. 8

In the current study, the UP was found to be of type 1 in ∼ 85% of the cases. Regarding direction, 81.9% of the UPs were medialized, 16.3% were vertical, 0.9% were lateralized, and 0.9% were absent, without significant differences in terms of gender and side.

The lateralization of the UP could obstruct the middle meatus and the ethmoidal infundibulum, which could lead to rhinosinusitis because the lateralized UP obstructs the maxillary ostium, preventing sinus ventilation.

We agree with Stammberger, 11 who considers that lateral UP placement could narrow the maxillary sinus ostium and lead to mucociliary clearance compromise, predisposing to sinus drainage blockage. Thus, the lateralized UP should be taken into consideration during ESS to avoid injury to the lamina papyracea and orbit. Also, Saunders et al. 18 found that rhinosinusitis presents more in lateralized Ups, and it is a factor in recurrent sinusitis and headache, due to its bad influence on sinus ventilation. 13

In the present study, pneumatization of the UP was detected in 8.63% of the subjects, without significance differences between genders. A lower prevalence of pneumatization of the UP was noticed in the studies by Shalini and Gopal 19 (4%), Srivastava and Tyagi 20 (1.6%), and Tuli et al. 21 (4%), while a higher prevalence was reported by Kumar et al. 22 (13%) and Ahmmed 23 (14.65%).

The present study showed that the pneumatization of the UP was associated with hyperpneumatization in the middle meatal area in 73.6% of the cases (Haller cell and concha bullosa), so once UP pneumatization is detected, the surgeon should search for other types of pneumatization and vice versa.

Pneumatization of the UP has also been cited as one of the anatomical variations that could impair sinus ventilation, particularly in the anterior ethmoidal sinus, frontal recess, and infundibulum region. It is also implicated as a potential cause of recurrent rhinosinusitis and headache in certain patients. However, UP pneumatization is not a common variation.

Preoperative evaluation of the UP variations can decrease the intraoperative and postoperative complications during ESS by protecting vital structures, such as the lamina papyracea, orbit, and cranial base. This can also decrease the chances of recurring rhinosinusitis. Detailed anatomical analysis of the UP is now possible through CT. Clinically, these anatomical variants are important, because they are involved as possible causes of complications and recurring rhinosinusitis.

The present study provides basic knowledge on the detailed descriptions of the UP variations observed on CT and updates the orientation about the UP from a CT perspective to provide the radiologists and surgeons with more data for ESS. Reviewing the CT assessment of the UP herein presented might aid in the operative planning and approach choices for diseases involving this area and in the preparation of the instrument set for each case.

However, it is recommended that the radiologist and surgeon study the UP assessment and types herein presented for various ethnic groups and diseases.

## Conclusion

The present study updates the CT knowledge of the UP to create a common language and improve radiologists' and surgeons' data on the UP in order to perform an effective and safe ESS.
